# Exogenous Brassinosteroid Facilitates Xylem Development in *Pinus massoniana* Seedlings

**DOI:** 10.3390/ijms22147615

**Published:** 2021-07-16

**Authors:** Fuhua Fan, Zijing Zhou, Huijuan Qin, Jianhui Tan, Guijie Ding

**Affiliations:** 1 Institute for Forest Resources and Environment of Guizhou, Guizhou University, Guiyang 550025, China; zhouzijing15@163.com (Z.Z.); qinhuijuan1029@163.com (H.Q.); 2 Key Laboratory of Forest Cultivation in Plateau Mountain of Guizhou Province, Guizhou University, Guiyang 550025, China; 3 College of Forestry, Guizhou University, Guiyang 550025, China; 4 Timber Forest Research Institute, Guangxi Academy of Forestry, Nanning 530009, China; tanjianhui94@163.com

**Keywords:** brassinosteroids, wood formation, secondary xylem, phytohormones, secondary cell wall, *Pinus massoniana*

## Abstract

Brassinosteroids (BRs) are known to be essential regulators for wood formation in herbaceous plants and poplar, but their roles in secondary growth and xylem development are still not well-defined, especially in pines. Here, we treated *Pinus massoniana* seedlings with different concentrations of exogenous BRs, and assayed the effects on plant growth, xylem development, endogenous phytohormone contents and gene expression within stems. Application of exogenous BR resulted in improving development of xylem more than phloem, and promoting xylem development in a dosage-dependent manner in a certain concentration rage. Endogenous hormone determination showed that BR may interact with other phytohormones in regulating xylem development. RNA-seq analysis revealed that some conventional phenylpropanoid biosynthesis- or lignin synthesis-related genes were downregulated, but the lignin content was elevated, suggesting that new lignin synthesis pathways or other cell wall components should be activated by BR treatment in *P. massoniana*. The results presented here reveal the foundational role of BRs in regulating plant secondary growth, and provide the basis for understanding molecular mechanisms of xylem development in *P. massoniana*.

## 1. Introduction

Wood or secondary xylem is a vascular tissue not only for water conduction, but also for mechanical support. Wood formation involves a cascade of complex and dynamic biological processes, including cell expansion, secondary cell wall (SCW) deposition and programmed cell death (PCD) [1]. SCW deposition produces the wood biomass, which is mainly composed of lignin, cellulose and hemicellulose. Understanding the mechanisms of secondary xylem development could raise valuable information to further increase the production of wood biomass. One of the important insights is that phytohormones play key roles in the development of xylem [2,3]. The signal transduction pathways for some phytohormones are quite well-documented, such as brassinosteroid (BR), gibberellin (GA) and auxin, whereas the molecular mechanisms of their regulating xylem development are not fully revealed [3,4].

Earlier studies showed hormonal control of wood formation, and described synergistic or antagonistic interactions between hormones in regulating vascular development [5,6]. Numerous experiments using exogenous hormones have demonstrated the potential effects of triggering xylem differentiation and stem growth. Auxin is proven to be a preliminary regulator in regulating wood formation by facilitating polar auxin transport and biosynthesis [7,8]. GA treatment induces the tension wood formation, accompanied with enhancing auxin response and upregulating key genes involved in BR response [9,10]. Several studies have also identified BRs as essential regulators for wood formation [4,11], and demonstrated that BRs were interacted with auxin and GA signaling pathways in secondary growth and vascular development [12,13,14]. BR biosynthesis pathways are highly networked, involving series of enzymes and genes, and the key steps are mediated by cytochrome P450 monooxygenases [4,15]. BRs are perceived by a receptor complex, BRASSINOSTEROID INSENSITIVE 1 (BRI1) and BRI1-ASSOCIATED KINASE (BAK1), at the plasma membrane [16], resulting in the dissociation of a negative regulator, BRI1 KINASEINHIBITOR 1 (BKI1) [17,18]. Then, BRI1 and BAK1 initiate transphosphorylation, followed by a cascade of phosphorylation and terminating with the BRASSINOSTEROID INSENSITIVE 2 (BIN2) kinase [4]. BIN2 phosphorylation regulates the expression of BRASSINAZOLE-RESISTANT1 (BZR1) and BRI1-EMS-SUPPRESSOR 1 (BES1/BZR2), further activating the expressions of downstream genes, which can regulate xylem development [18,19,20].

Xylem development is a complex process that requires the expressions of a series of genes involved in biosynthesis, secretion, deposition and assembly of components to form SCW [3]. Previous studies have identified many genes involved in biosynthesis of SCW components, including xylan, glucomannan, cellulose, hemicellulose and lignin [21,22]. In addition, some transcriptional factors (TFs), such as NAC (no apical meristem (NAM), *Arabidopsis thaliana* transcription activation factor (ATAF1/2) and cup-shaped cotyledon (CUC2)), MYB (v-myb avian myeloblastosis viral oncogene homolog), WRKY, bHLH (basic helix-loop-helix) and AP2/ERF (APETALA2/ethylene response factor) family, also play an important role in regulating transcript accumulation of the SCW biosynthesis-related genes [23,24,25,26]. However, it remains far from complete elucidating the mechanisms of xylem development, especially in pines.

*Pinus massoniana* is a native coniferous gymnosperm in southern China. This species is widely used for afforestation, and considered to be an important economic tree for its resin, pulp and timber production [22]. Due to the lack of genomic information, transcriptome analyses based on high-throughput RNA-sequencing have been widely used in *P. massoniana* to acquire genomic information or identify target genes. A previous study identified many lignin biosynthesis-related genes of *P. massoniana* through comparative transcriptome studies with materials of different diameter at breast height using RNA-seq technology [22]. Here, we present experiments in *P. massoniana* showing a positive role of exogenous BR in promoting xylem development. Endogenous phytohormone levels were detected to determine if the BR functioned coordinately or antagonistically with GAs and auxin in regulating stem growth. Wood formation-related genes were identified through RNA-seq. The results provide insights into the roles of BR in stem growth and contribute to our understanding of the molecular mechanisms of xylem development in pines.

## 2. Results

### 2.1. BR Treatment Facilitates Stem Growth and Xylem Development during Secondary Growth

**Figure 1:**
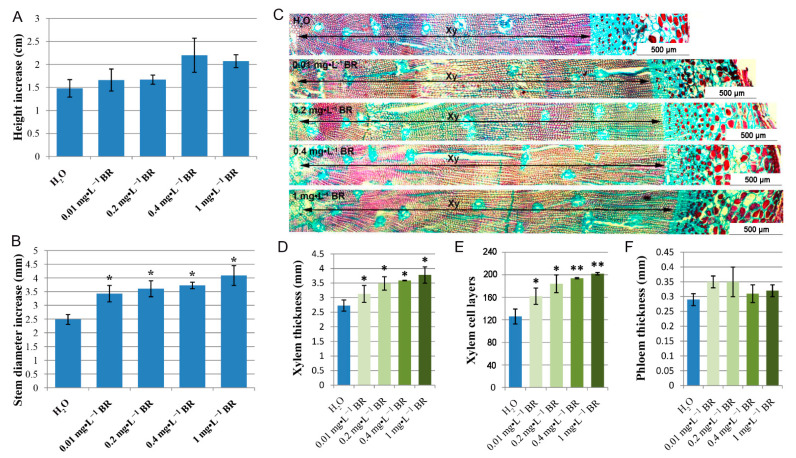
Effects of exogenous BR on stem growth and xylem development after four-month growth at series BR treatment. (**A**) Quantification of height increase of seedlings. (**B**) Stem diameter increase at series BR treatment. (**C**) Normal histology of stem sections. Bar = 500 µm. Xy, xylem. (**D**) Xylem thickness at series BR treatment. (**E**) Number of xylem cell layers at series BR treatment. (**F**) Phloem thickness at series BR treatment. Error bars ± SE (standard error). * and ** indicate significant differences in comparison with control at *p* < 0.05 and *p* < 0.01, respectively.

To determine whether exogenous BR affect normal growth and xylem development of *P. massoniana*, seedlings were treated with spray-applied BR for four months with different concentrations. Each seedling was measured for plant height, basal diameter and anatomical features. As shown in Figure 1A,B, treatment of seedlings with exogenous BR resulted in a greater increment of plant height and stem diameter for all concentrations of BR compared to control. Furthermore, the stem diameter significantly increased in a BR dosage-dependent manner in this study. Seedlings treated with 1 mg·L^−1^ BR attained the greatest increment of basal diameter. Histological comparison showed that xylem thickness and the number of xylem cell layers improved significantly in BR-treated seedlings (Figure 1C–E), while no significant differences were observed for phloem (Figure 1F). In addition, the xylem thickness and cell layer number increased with the increase of BR concentration.

### 2.2. Effect of Exogenous Application of BR on the Contents of Lignin, Cellulose and Hemicellulose

**Figure 2:**
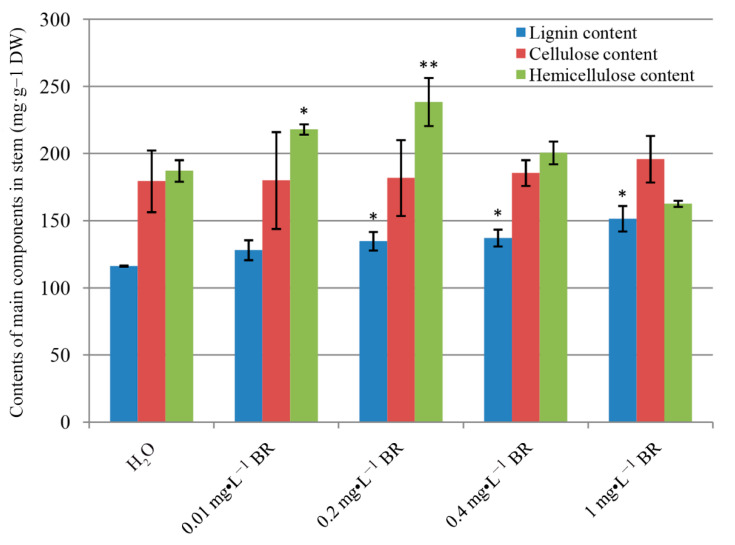
Quantification of the contents of lignin, cellulose and hemicellulose after four-month growth at series of BR treatment. DW means drought weight. Error bars ± SE. * and ** indicate significant differences in comparison with control at *p* < 0.05 and *p* < 0.01, respectively.

Stems of *P. massoniana* seedlings treated with BR were collected and analyzed for the contents of lignin, cellulose and hemicellulose to investigate the role of BR on wood formation. Exogenous application of BR altered wood formation and property (Figure 2). The lignin content was significantly dosage-dependently increased after four months of BR treatment. In the control group, the lignin content was 116.31 mg·g^−1^, subsequently raised after BR treatment, and reached a maximum of 151.54 mg·g^−1^ at 1 mg·L^−1^ BR, an increase of 30.29%. The cellulose content was slightly increased for all concentrations of BR compared with control, but not significantly. Whereas, the hemicellulose content significantly improved, subsequently declined with the increase of BR concentration, and the maximum value reached 238.43 mg·g^−1^ at 0.2 mg·L^−1^ BR, an increase of 27.41%, compared with control. It is worth noting that the hemicellulose ratio was the highest among the three components under 0–0.4 mg·L^−1^ BR treatment, while the cellulose ratio reached the maximum at 1 mg·L^−1^ BR.

### 2.3. Changes of Endogenous Phytohormone Levels in Seedling Stems in Response to BR Treatment

**Figure 3:**
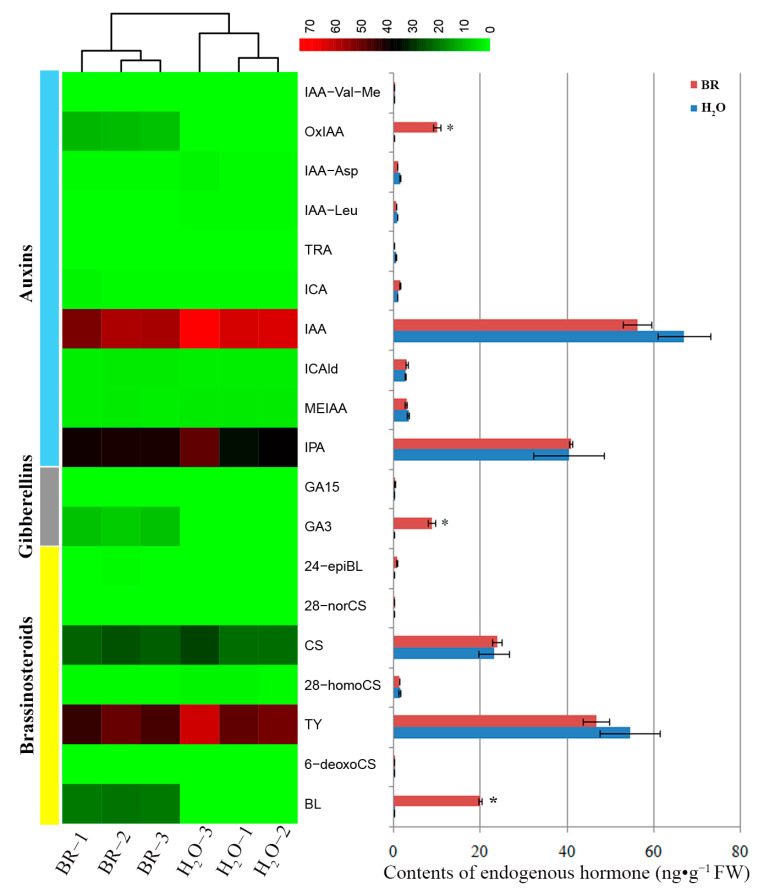
Endogenous hormone levels of the seedling stems after 1 mg·L^−1^ BR and H_2_O treatment. FW means fresh weight. Error bars ± SE. * indicates significant differences in comparison with control at *p* < 0.05. The color brightness represents the degree of the content difference, as shown in the color bar. IAA-Val-Me: indole-3-acetyl-L-valine methyl ester; OxIAA: 2-oxindole-3-acetic acid; IAA-Asp: indole-3-acetyl-L-aspartic acid; IAA-Leu: N-(3-Indolylacetyl)-L-leucine; TRA: tryptamine; ICA: indole-3-carboxylic acid; IAA: indole-3-acetic acid; ICAld: indole-3-carboxaldehyde; MEIAA: methyl indole-3-acetate; IPA: 3-indolepropionic acid; GA15: gibberellin A15; GA3: gibberellin A3; 24-epiBL: 24-epibrassinolide; 28-norCS: 28-norcastasterone; CS: castasterone; 28-homoCS: 28-homocastasterone; TY: typhasterol; 6-deoxoCS: 6-deoxocastasterone; BL: brassinolide.

The above results indicated that 1 mg·L^−1^ BR treatment resulted in maximal growth of xylem. To determine the effect of exogenous BR on the changes of phytohormones, endogenous hormone (including auxins, GAs and BRs) levels were assayed in 1 mg·L^−1^ BR-treated and control samples (Figure 3). The results showed that the levels of 2-oxindole-3-acetic acid (OxIAA), gibberellin A3 (GA3) and brassinolide (BL) were significantly elevated in the stems of 1 mg·L^−1^ BR-treated seedlings compared with controls. Indole-3-acetic acid (IAA) content was slightly declined compared with H_2_O treatment, but it remained at a relatively high level. There were no significant changes in the levels of other hormones between BR-treated seedling stems and control.

### 2.4. Transcription Level of Genes Associated with Xylem Development in Response to BR Treatment

To further study the mechanism of how BR affects wood formation, RNA-seq was performed to quantify the expressed genes in both stems of 1 mg·L^−1^ BR-treated seedlings of *P. massoniana* and control after four-month treatments. The RNA-seq data for all samples are available on line http://www.ncbi.nlm.nih.gov/sra (accessed on 8 March 2021) under accession number PRJNA707606.

**Figure 4:**
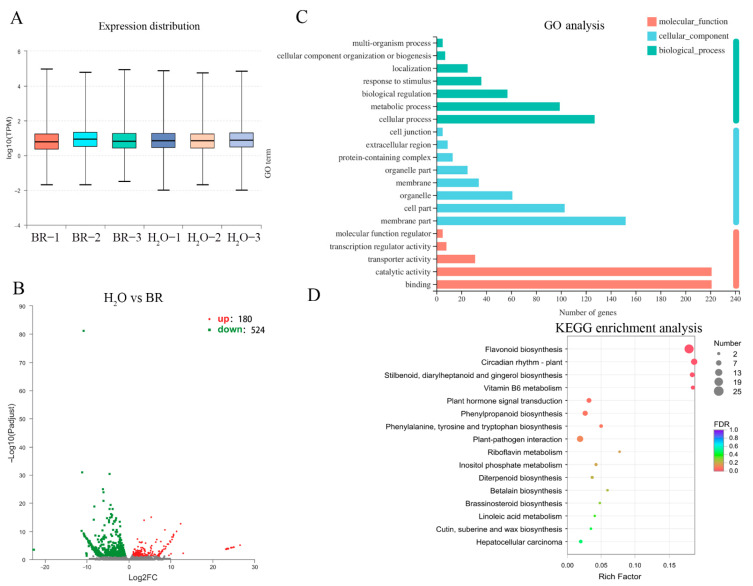
Identification of relative differentially expressed genes (DEGs) after 1 mg·L^−1^ BR treatment and enrichment analysis of unigenes. (**A**) Unigene expression abundance of each sample. (**B**) Identification of DEGs between 1 mg·L^−1^ BR and H_2_O treatment. Red and green dots indicate the up- and down-regulated genes, respectively. (**C**) Top 20 of GO enrichment analysis of DEGs for cellular component, biological process and molecular function. (**D**) KEGG enrichment of DEGs. The size of dots represents the gene number, and color of dots represents the FDR value.

Gene transcript abundance was quantified (Figure 4A) and the data were used to identify the DEGs by DEGseq, with |log2FC| > 1 and FDR ≤ 0.05, resulting in a total of 704 identified DEGs, including 180 upregulated and 524 downregulated genes (Figure 4B, Supplementary Table S1). To fully explore the function of DEGs, 704 DEGs were characterized into 39 GO terms of 3 categories (cellular component, biological process and molecular function) (Figure 4C). The top two enriched GO terms were “binding” and “catalytic activity” in the category of molecular function, followed by “membrane part” (47 DEGs) of the cellular component and “cellular process” of the biological process. The KEGG enrichment analysis showed that 191 DEGs were characterized into 66 pathways in the KEGG database (Figure 4D). The top five most significantly enriched pathways were “flavonoid biosynthesis”, “circadian rhythm-plant”, “stilbenoid, diarylheptanoid and gingerol biosynthesis”, “vitamin B6 metabolism” and “plant hormone signal transduction”.

**Figure 5:**
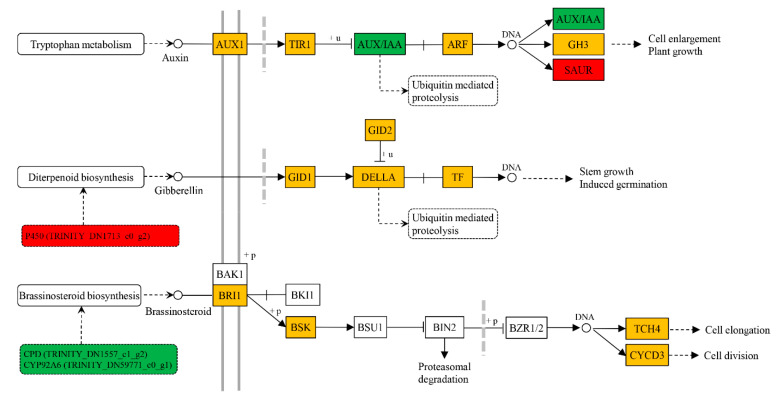
Endogenous hormone biosynthesis and signal transduction of auxin, giberellin and brassinosteroid. +u and +p mean ubiquitination and phosphorylation, respectively. Red and green frames indicate significantly upregulated and downregulated, respectively. Orange frame means gene expression was induced, but not significantly.

Exogenous BR changed the content of endogenous hormones (Figure 3), meanwhile, it influenced the gene expressions associated with biosynthesis, metabolism and signal transduction of endogenous hormones (Figure 5, Table 1). The cytochrome P450 genes, *constitutive photomorphogenesis* and *dwarf* (*CPD*, TRINITY_DN1557_c1_g2) and *dark-induced DWF-like protein 1* (*DDWF1/CYP92A6*, TRINITY_DN59771_c0_g1) regulating brassinosteroid biosynthesis were significantly repressed after exogenous BR treatment. Even so, the membrane receptor, *BRASSINOSTEROID-INSENSITIVE 1* (*BRI1*), was slightly induced for exogenous BR application. A multifunctional oxidase gene in diterpenoid and gibberellin biosyntheses encoding a cytochrome P450 (TRINITY_DN1713_c0_g2) was significantly upregulated in the BR-treated seedlings. A gibberellin-regulated protein gene (*GRP*, TRINITY_DN7994_c0_g1) was also significantly induced by exogenous BR. Even if no inducible expression of auxin biosynthesis-related genes was demonstrated in this study, OxIAA content was improved by exogenous BR (Figure 3), and an auxin-responsive protein gene *small auxin upregulated RNA* (*SAUR*, TRINITY_DN8347_c0_g1) showed significant upregulation, as well as auxin polar transport-associated gene (*APT-like*, TRINITY_DN68002_c0_g2), whereas another auxin-responsive protein gene *auxin/indole-3-acetic acid* (*AUX*/*IAA*, TRINITY_DN1260_c0_g1) was downregulated. An important gene negatively regulating accumulation of the plant BR, *lateral organ boundaries* (*LOB*, TRINITY_DN142222_c0_g1), was repressed in the BR-treated stems.

Wood formation is due to xylem differentiation and secondary cell wall thickening, resulting from the biosynthesis and deposition of lignin, cellulose and hemicellulose. Lignin biosynthesis-related genes *hydroxycinnamoyl-CoA shikimate/quinate hydroxycinnamoyl transferase* (*HCT*, TRINITY_DN10676_c0_g1) and *catechol O-methyltransferase* (*COMT*, TRINITY_DN48043_c1_g2) were significantly upregulated, but *phenylalanine ammonia-lyase* (*PAL*, TRINITY_DN40525_c0_g1), *cinnamic acid 4-hydroxylase* (*C4H*, TRINITY_DN4395_c0_g1), *cinnamyl-alcohol dehydrogenase* (*CAD*, TRINITY_DN16664_c0_g1) and *peroxidase* (*PER*, TRINITY_DN21453_c0_g2, TRINITY_DN8974_c0_g1, TRINITY_DN149552_c0_g1) showed significant misregulation in this study. Cellulose biosynthesis-related gene *cellulose synthase* (*CESA*, TRINITY_DN12265_c0_g1) was also downregulated after 1 mg·L^−1^ BR treatment for four months. Meanwhile, the expressions of other orthologous genes involved in cell wall biosynthesis, such as *choline/ethanolamine kinase* (*CHK*, TRINITY_DN1519_c0_g1), 3-beta hydroxysteroid dehydrogenase/isomerase (*3Beta-HSD*, TRINITY_DN10086_c0_g1), *pectate lyase* (*Pel*, TRINITY_DN11956_c0_g5), *glycosyl transferase* (*GT*, TRINITY_DN9779_c0_g5) and *xyloglucan endotransglycosylase* (*XET*, TRINITY_DN10198_c0_g1), were altered under BR treatment (Table 2 and Supplementary Table S1). Similar to other biological processes, xylem development is controlled by TFs. The expression levels of some TFs, such as *AP2/EREBP* (TRINITY_DN89065_c0_g1), *ERF18* (TRINITY_DN7860_c0_g1), *WRKY* (TRINITY_DN1793_c0_g1) and *PPR* (TRINITY_DN11063_c0_g1), were significantly increased, which may be the regulators for xylem development.

### 2.5. RT-qPCR Validation of DEGs from the RNA-Seq

**Figure 6:**
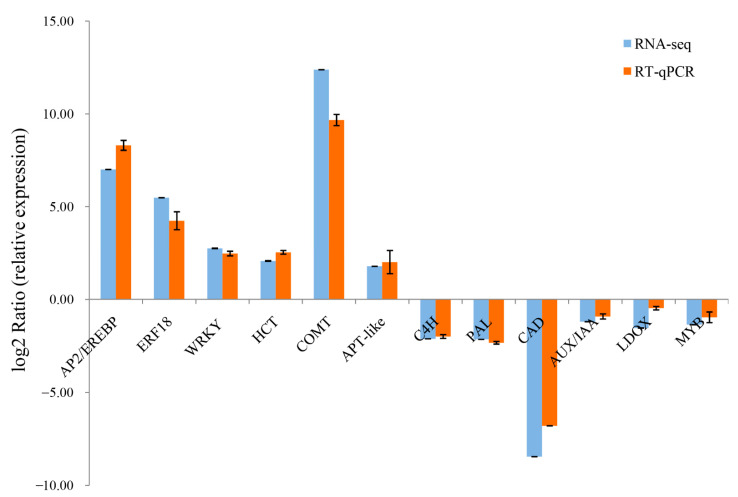
RT-qPCR validation of selected unigenes involved in xylem development. The blue and orange bars represent the normalized relative expression determined by RNA-seq and RT-qPCR, respectively. Error bars ± SE.

To validate the expression pattern of hormone-responsive genes and xylem development genes screened from the RNA-seq, the expression levels of these related genes were examined using RT-qPCR (Figure 6). The results showed that there was consistency between the RNA-seq analyses and the RT-qPCR results for all selected genes, which confirmed the reproducibility and reliability of the transcriptome data.

## 3. Discussion

BR is recognized to have a positive role in stimulating xylem development [18]. Application of exogenous BR or increasing endogenous BR levels promoted vascular cell differentiation and secondary xylem formation [11,27]. In the present study, significant increases in xylem thickness and xylem cell layers were observed in BR-treated plants. In addition, the xylem thickness, cell layer number and lignin content were increased with the increase of BR concentration (Figure 1 and Figure 2), suggesting that exogenous BR promoted xylem development in a dosage-dependent manner in a certain concentration rage. A more detailed analysis of endogenous phytohormone (including auxins, GAs and BRs) levels showed that BL, GA3 and OxIAA were elevated in the stems after BR treatment, whereas IAA level was slightly declined. These results suggest that BR may interact synergistically with GA3 and antagonistically or independently with IAA in promoting *P. massoniana* xylem development.

BR signaling components might promote stem growth by cell elongation and accelerating cell division [3,28]. Our results revealed that exogenous application of BR suppressed the expression of BR-synthesis-related genes (*CPD* and *CYP92A6*). However, the BL level in stems was elevated, and downstream genes of BR signaling were induced. *TCH4*, encoding a xyloglucan endotransglycosylase, is proven to be involved in regulating cell elongation and induced by BR with low kinetics [29]. Another BR-stimulated gene, *cyclinD3* (*CycD3*), shows a promotive effect on cell division [30]. In plants, BRs and auxin activate cell expansion through interdependent mechanisms [13,31]. Our results showed that exogenous BR improved the OxIAA level but slightly decreased the IAA level, and the total auxin levels did not seem to change. RNA-seq analysis revealed that BR treatment did not increase the expression of auxin biosynthesis-related genes, confirming that BRs do not regulate auxin biosynthesis [31]. As key players in auxin-signal transduction, *AUX/IAA* genes are involved in BR responses in a manner dependent on organ type [32]. In this study, an *AUX/IAA* gene was repressed by exogenous BR in *P. massoniana* stems, but another auxin-responsive gene, *SAUR*, was induced. SAURs play a central role in plant growth and development [33]. Our results suggested that BR stimulates *SAUR* transcription independently of auxin level changes, and that it is important in BR-mediated cell elongation. Recently, studies reported that BR induces gene expressions for GA biosynthesis, resulting in improvement of GA levels [34,35,36]. A putative cytochrome P450 gene involved in diterpenoid or gibberellin biosynthesis was induced, and the content of GA3 was improved in this study, suggesting the coordinate regulation of BR and GA in xylem development.

Exogenously applied BR might reduce lignification and increase fiber cell types in plant stems [37]. However, in this study, cellulose content of seedling stems was slightly affected by BR treatments, and the effects were not significant (*p* > 0.05). The hemicellulose content was declined with excessive BR concentration, whereas lignin content of seedling stems was elevated after BR treatments. These results conflict with previous studies showing that BR significantly upregulated cellulose biosynthesis and repressed lignin deposition [37,38], while the results of this work are consistent with a study of switchgrass, showing that BR application increased amounts of lignin [39]. Taken together, BR effects on cell wall components may differ in different plants or organs, but it does affect cell wall integrity in plants [3]. RNA-seq analysis in this study revealed that a cellulose biosynthesis-related gene, *CESA*, was slightly downregulated by BR treatment, but several carbohydrate-related genes involved in cell wall biosynthesis were induced (Table 2). As one of the major components of wood, lignin is traditionally described as being composed of *p*-hydroxyphenyl (H-lignin), guaiacyl (G-lignin) and syringyl (S-lignin) units, and G-lignin is mainly contained in gymnosperms [22,40]. Lignin biosynthesis is believed to be synthesized from phenylalanine [22], but no phenylalanine biosynthesis-related genes were upregulated in this study. Most phenylpropanoid biosynthesis- or lignin biosynthesis-related genes (such as *PAL*, *C4H*, *CAD* and *PER*) were downregulated after BR treatment, except for *HCT* and *COMT*. These results seem to conflict with the conclusion of the physiological index. Recent studies have revealed that lignin in plant stems is not only synthesized from phenylalanine but also from tyrosine [41]. There was a tyrosine metabolism-related gene that was dramatically induced in this study (Table 2), suggesting the different esters pathway to monolignols in *P. massoniana*. HCT can act as a ‘reverse’ reaction to form caffeoyl CoA [41], and plays a key role in accumulation of G-lignin [42]. COMT is transitionally recognized as a key enzyme in S-lignin synthesis [22]. Recently, it was proven to methylate coumarate 3-hygroxylase (C3H) to ferulic acid [43], and this process was supposed to reinstate the synthesis pathway of G-lignin [41].

Plant cell walls also contain many other polysaccharide and glycoprotein components [44]. Although it is difficult to identify the role of TFs in the synthesis of specific components, SCW formation or development is known to be regulated by a cascade of TFs. Here, we found that the expressions of some TFs positively correlate with xylem thickness and cell layers, suggesting that these TFs play a role in xylem development. Ethylene-related TFs have been shown to promote cambial growth and cell differentiation [45]. Recently, AP2/EREBP domain transcription factors have been reported to participate in plant secondary wall formation [3]. Overexpression of *ERF18* in hybrid aspen modified the wood chemotype and enhanced diameter growth [46]. Our results revealed that *AP2/EREBP* and *ERF18* exhibited about 6- and 44-fold increased expression levels respectively, in response to BR treatments. It has been suggested that ethylene-responsive transcription factors may be key transcription factors that coordinately regulate xylem development in *P. massoniana*. Most WRKYs function as repressors of pith secondary wall formation [47]. More recently, AtWRKY13 has been shown to positively regulate the development of stem [48]. In this study, putative *WRKY* were induced by exogenous BR. We supposed that *WRKY* might be involved in hormone signaling and modulate xylem development.

## 4. Materials and Methods

### 4.1. Plant Materials and Growth Conditions

Two-year-old container seedlings of *P. massoniana* “Fu 5” (collected from Duyun Forest Farm of Guizhou, Guiyang, China) were used in all experiments. The seedlings were planted in 3.5 L pots containing a mixture of vermiculite and nutrient soil. Aerial surfaces of seedlings were sprayed every 7 days for 4 months with different concentrations of exogenous brassinolide (0.01, 0.2, 0.4 and 1 mg·L^−1^). The same volume of ultrapure water was applied as a control treatment. After four months of treatment, stems of seedlings were harvested for histological, physiological and RNA-sequencing analyses, and three samples were taken from each treatment as biological replicates for each experiment.

### 4.2. Morphology, Histology and Imaging

Seedling height and stem diameter at base were measured using tape and vernier caliper. Fresh stems from comparable areas were fixed with formaldehyde-acetic acid-alcohol (FAA) solution for 48 h, softened in 70% tert-butanol/glycerol (1:1, *v*/*v*) solution, dehydrated through a series of graded ethanol solutions and subsequently vitrified using dimethylbenzene. Then, samples were embedded into paraffin, and sectioned using a Leica RM2235 rotary microtome (Leica, Wetzlar, Germany). The sections were stained with 1% safranin and 1% fast green and examined using a Leica DM2500 microscope (Leica, Wetzlar, Germany) after removing paraffin. Radial widths of xylem and phloem were measured using Leica Application Suite X 3.0.2 (Leica, Wetzlar, Germany).

### 4.3. Quantitative Determination of Lignin, Cellulose and Hemicellulose

Approximately 2 mg of oven-dried stem powder was homogenized using 80% (*v*/*v*) ethanol and heated in a water bath at 85 °C for 10 min. After centrifugation at 12,000 rpm for 10 min, the sediment was collected for quantifying lignin, cellulose and hemicelluloses. Based on acetylation of lignin phenolic hydroxyl group, lignin content was determined using a kit (G0708W48, Suzhou Grace Bio-technology Co. LTD, Suzhou, China) according to the manufacturer’s instructions. For determination of cellulose content, the kit (G0715W48, Suzhou Grace Bio-technology Co. LTD, Suzhou, China) was used on the basis of hydrolysis and dehydration of cellulose into furfural compounds, which can react with anthrone. The content of hemicellulose was determined by colorimetry to determine the xylose content generated by hydrolysis of hemicelluloses using the kit (G0716W48, Suzhou Grace Bio-technology Co. LTD, Suzhou, China).

### 4.4. Analysis of Phytohormones Levels by LC-MS/MS

To analyze the responsiveness of phytohormones (including auxins, GAs and BRs) with BRs treatment on wood formation, 80 mg fresh segments were obtained from the basal stems of control and BR-treated seedlings. For BRs concentration measurements, samples were ground into powder under liquid nitrogen and extracted with 1 mL of HPLC-grade acetonitrile (CAN). The combined solutions were vortexed, incubated at 75 °C for 1 h and evaporated to dryness under nitrogen gas stream, followed by redissolving in 100 µL of CAN. For auxins and GAs concentration measurements, samples of powder were extracted with 1 mL of methanol/water/formic acid (15:4:1, *v*/*v*/*v*), and the dried extracts were reconstituted in 100 μL of 80% methanol (*v*/*v*). Then, the reaction solutions were filtered through a 0.22 μm filter for further LC-MS analysis. Phytohormones contents were detected by MetWare (Wuhan, China) based on the AB Sciex QTRAP 6500 LC-MS/MS platform.

### 4.5. RNA Isolation, cDNA Library Construction and RNA-Seq

Total RNA was extracted from the stem using plant TRIzol^®^ Reagent based on the manufacturer’s instructions (Invitrogen, Carlsbard, CA, USA) and genomic DNA was removed using DNase I (TaKara). The quality and quantity of the total RNA was assessed by a 2100 Bioanalyzer (Agilent Technologies, Santa Clara, CA, USA) and ND-2000 (NanoDrop Thermo Scientific, Wilmington, DE, USA), separately. Library construction and sequencing were performed at Shanghai Majorbio Bio-pharm Biotechnology Co., Ltd. (Shanghai, China), according to the manufacturer’s instructions (Illumina, San Diego, CA, USA). Poly (A) mRNA was specifically purified from the total RNA with oligo (dT)-attached magnetic beads and fragmented by fragmentation buffer. Then, double-stranded cDNA was synthesized using a SuperScript double-stranded cDNA synthesis kit (Invitrogen, Carlsbad, CA, USA) with random hexamer primers (Illumina), and subjected to end-repair, phosphorylation and ‘A’ base addition according to Illumina’s library construction protocol. The ligation products were size selected for cDNA target fragments of 200–300 bp by 2% Low-Range Ultra Agarose followed by PCR amplification, quantification and sequencing using an Illumina NovaSeq 6000 sequencer (Illumina, San Diego, CA, USA) for 2 × 150 bp paired-end reads.

### 4.6. Data Processing and Bioinformatics Analysis

The raw paired-end reads were trimmed and quality-controlled by SeqPrep (https://github.com/jstjohn/SeqPrep) and Sickle (https://github.com/najoshi/sickle) with default parameters. Then, high-quality clean reads were selected for de novo assembly with Trinity (http://trinityrnaseq.sourceforge.net/). For homology annotation, the assembled transcripts were searched against the NCBI protein non-redundant (NR), Clusters of Orthologous Groups (COG) and Kyoto Encyclopedia of Genes and Genomes (KEGG) databases using BLASTx, with a typical cut-off E-value less than 1.0 × 10^−5^.

The expression level of each transcript was calculated according to the transcripts per million reads (TPM) method. Gene abundances were quantified by RSEM (http://deweylab.biostat.wisc.edu/rsem/), and differentially expressed gene (DEG) analysis was performed using the DEGseq, with |log2FC| > 1 and false discovery rate (FDR) ≤ 0.05 as the threshold. In addition, functional-enrichment analysis was performed by Gene Ontology (GO) and KEGG at Bonferroni-corrected *p*-value ≤ 0.05 compared with the whole-transcriptome background. GO enrichment analysis provides all GO terms that are significantly enriched in DEGs for describing biological processes, molecular functions and cellular components. The KEGG algorithm is utilized to identify significantly enriched metabolic pathways or signal transduction pathways.

### 4.7. Validation of Gene Expression Profiles via RT-qPCR

Twelve differentially expressed unigenes were randomly selected and quantitatively analyzed using RT-qPCR to verify RNA-seq results. Primers were designed using Primer Premier 5.0 (Supplementary Table S2). The Talent qPCR PreMix (SYBR Green) Kit (Tiangen, Beijing, China) was used for triplicate RT-qPCR reactions with a CFX96 Touch Real-Time PCR System (Bio-Rad, Hercules, CA, USA) based on provided directions. The cycle threshold (Ct) and 2^−ΔΔCt^ method were utilized to assess relative transcript levels for each gene, which were normalized using *UBC* as internal controls.

### 4.8. Statistical Analysis

The statistical analysis of the BR-treated and control sample phenotypes and RT-qPCR data were performed using SPSS 18.0 software (SPSS, Chicago, IL, USA). Analyses of variance (ANOVA) for sets of data were analyzed with Duncan’s testing method to determine differences between pairs of means of multiple experiments. *p* < 0.05 and *p* < 0.01 were considered to be significant and extremely significant, respectively.

## 5. Conclusions

Our results indicated that exogenous BR appeared to affect development of xylem more than phloem, and promoted xylem development in a dosage-dependent manner in a certain concentration rage. It is critical that BR may interact with other phytohormones in regulating xylem development, because the levels of OxIAA, IAA, GA3 and BL were significantly changed in the stems of BR-treated seedlings compared with controls. RNA-seq analysis revealed that some conventional phenylpropanoid biosynthesis-related genes, such as *PAL*, *C4H*, *CAD* and *PER*, were downregulated, whereas other cell wall biosynthesis-related genes, such as *Pel*, *GT* and *XET*, were altered under BR treatment, suggesting that a new lignin synthesis pathway or other cell wall components should be activated by BR treatment. This study provided a revealing insight into the xylem development of *P. massoniana* seedlings in response to BR signaling.

## Figures and Tables

**Table 1:** Important genes in biosynthesis, metabolism and signal transduction of endogenous hormones.

Functional Classification	Gene Name	Accession Number	Description	Fold Change Log2 (BR/Control)
Phytohormone biosynthesis	*CPD*	TRINITY_DN1557_c1_g2	Cytochrome P450, oxidoreductase activity	−2.35
	*CYP92A6*	TRINITY_DN59771_c0_g1	Cytochrome P450, monooxygenase activity	−7.13
Phytohormone metabolism and signal transduction	*GRP*	TRINITY_DN7994_c0_g1	Gibberellin-regulated protein, posttranslational modification, protein turnover, chaperones	4.69
	*DAO*	TRINITY_DN9023_c0_g1	Gibberellin 3-beta-dioxygenase activity	−2.81
	*LOB*	TRINITY_DN142222_c0_g1	Lateral organ boundaries (LOB) domain-containing protein, response to gibberellin	−3.73
	*APT-like*	TRINITY_DN68002_c0_g2	Regulation of auxin polar transport	1.79
	*SAUR*	TRINITY_DN8347_c0_g1	Auxin responsive family-like protein	2.21
	*5NG4*	TRINITY_DN370_c0_g1	Auxin-induced protein	−1.31
	*AUX/IAA*	TRINITY_DN1260_c0_g1	Auxin-responsive protein IAA7	−1.19

**Table 2:** Important upregulated genes in biosynthesis and metabolism of cell wall components.

Functional Classification	Gene Name	Accession Number	Description	Fold Change Log2 (BR/Control)
Phenylpropanoid biosynthesis	*HCT*	TRINITY_DN10676_c0_g1	Agmatine coumaroyltransferase	2.07
Tyrosine metabolism	*COMT*	TRINITY_DN48043_c1_g2	Catechol O-methyltransferase A	12.38
Glycerophospholipid metabolism	*CHK*	TRINITY_DN1519_c0_g1	Choline/ethanolamine kinase	1.89
Carbohydrate transport and metabolism	*3Beta-HSD*	TRINITY_DN10086_c0_g1	3-beta hydroxysteroid dehydrogenase/isomerase	1.47
	*UDPGT*	TRINITY_DN18407_c0_g2	UDP-glucoronosyl and UDP-glucosyl transferase	5.18
	*PIP*	TRINITY_DN8514_c0_g1	Probable aquaporin PIP2-6	3.43

## Data Availability

The data presented in this study are available on request from the corresponding author.

## Supplementary Materials

The following are available online at https://www.mdpi.com/article/10.3390/ijms22147615/s1. The RNA-seq data for all samples are available at the Sequence Read Archive of the National Center for Biotechnology Information (http://www.ncbi.nlm.nih.gov/sra, accessed on 8 March 2021) under accession number PRJNA707606.
